# Factors influencing fever care-seeking for children under five years of age in The Gambia: a secondary analysis of 2019–20 DHS data

**DOI:** 10.1186/s12936-024-04951-w

**Published:** 2024-04-27

**Authors:** Laura Arntson, Katherine R. McLaughlin, Ellen Smit

**Affiliations:** 1 Oregon State University College of Health, 160 SW 26th St, Corvallis, OR 97331 USA; 2 Department of Statistics, Oregon State University, 239 Weniger Hall, Corvallis, OR 97331 USA

**Keywords:** Child fever, Malaria, Care-seeking, Decision-making

## Abstract

**Background:**

Malaria contributes to excess child mortality in The Gambia. Children under five are at risk of severe malaria and death if not treated promptly and appropriately. It is crucial that a child with fever receive appropriate care from a trained provider. The aim was to identify influences on child fever care-seeking in The Gambia to inform malaria control strategies.

**Methods:**

This cross-sectional analysis of The Gambia 2019–20 Demographic and Health Survey used logistic regression analysis to identify associations between source of care for a child with fever (public or private healthcare provider, other, or no treatment) and mother, child, and household characteristics.

**Results:**

Only 52.0% of mothers sought care from a trained healthcare provider for a child with fever—45.1% from a public facility and 7.0% from the private sector. 35.2% of mothers did not seek treatment. Mothers in urban households were 2.67 times as likely (aOR, 95% CI 1.504–4.736) as mothers in rural households to seek care from an informal source (e.g., pharmacy) versus not seeking treatment, and 0.29 times as likely (aOR, 95% CI 0.165–0.515) as mothers in rural households to seek care from a public provider versus informal source. Mothers in wealthier households were 2.30 times as likely (aOR, 95% CI 1.274–4.164) as mothers in poorer households to seek care from an informal source versus no treatment and half as likely as mothers in poorer households to seek care from a public provider versus informal source (aOR 0.53, 95% CI 0.291–0.959).

**Conclusions:**

Maintaining The Gambia’s malaria control achievements will require the active engagement and oversight of private pharmacies along with continued integrated community case management to reach mothers who do not seek care for a child with fever, and remove challenges to seeking appropriate care from trained providers. Whether influenced by convenience, costs, perceived urgency, or other factors, given the likelihood of urban mothers and mothers in wealthier households to seek care from private pharmacies, it will be necessary to incorporate private pharmacies into malaria control strategies while building public sector capacity and workforce, and initiating more effective attitude and behavioural change among mothers and households.

## Background

Children under five years of age accounted for 80.0% of all deaths attributed to malaria in the WHO African region between 2019 and 2020 [1]. Malaria is among the leading causes of under-five mortality in The Gambia. The Gambia 2018 multiple indicator cluster survey reported deaths among children under five attributable to malaria at 7.0% [2]. Children under five in endemic malaria regions are at risk of developing high *P. falciparum* malaria parasite densities that can quickly progress to severe malaria, which, if not treated promptly and appropriately can lead to death [3–6]. It is crucial that children under five with fever in malaria endemic regions receive appropriate care, including diagnostic testing and treatment or referral from a trained provider [6–11].

The Gambia has made significant progress in reducing overall malaria prevalence [12] and incidence. Between 2015 and 2019, malaria incidence in The Gambia decreased from 199.9 to 43.8 per 1000 population at risk [13]. To ensure continued progress toward the country’s goal of eliminating malaria [14], The Gambia must address the challenges mothers face in seeking appropriate care for a child with fever.

In spite of numerous attempts to understand child fever care-seeking behaviour in sub-Saharan Africa, the predictors of care-seeking behaviour are still not well understood (15–17). Residence [18–21], household wealth, socio-economic status, or poverty [22–24], distance or access to a facility or provider [17, 25–27], a lack of women’s empowerment or autonomy, [28, 29] and the social dynamics of decision-making [30] have been addressed in the literature, yet the interplay of various factors and their influence on care-seeking by type of source of care remains somewhat opaque.

This study builds on previous studies to address this knowledge gap by bringing themes from qualitative and quantitative research on care-seeking into a discussion of the patterns observed in an analysis of demographic and health survey (DHS) data, toward identifying competing challenges and incentives that mothers face in seeking care and treatment for a child with fever in The Gambia. Care-seeking decision-making does not adhere to normative decision theory [31]. It is a process that reflects an oscillation between order and disorder in the “restless processuality of social life” [32]. Decisions reflect internal beliefs and uncertainty as well an individual’s social ecology, in which household power dynamics and gendered spaces of activity can dictate perceptions of options for economic and health behaviour [33]. Among competing incentives are: convenience of access to care and treatment given residence, distance, costs, and negotiating household priorities and decision-making in the household [34–36].

## Methods

### Study design and data collection

This was a cross-sectional study of The Gambia 2019–20 Demographic and Health Survey (DHS) child dataset [37]. This study used multinomial logistic regression analysis to examine the association between: household wealth, other household characteristics, and mother and child characteristics; and the type of source of care-seeking for a child with fever. The Gambia 2019–20 DHS data collection was completed between November 21, 2019 and March 30, 2020 [2]. The child dataset was generated by linking data from the DHS household questionnaire, woman’s questionnaire, man’s questionnaire, and a biomarker questionnaire [38].

### Selection of participants and survey response rates

The Gambia 2019–20 DHS used a stratified 2-stage cluster design with probability of selection proportional to size within each sampling stratum for a total sample size of 7025 households, representative of the resident population. All women ages 15–49 who were either permanent residents of a selected household or visitors who stayed in a selected household the night before the survey were eligible to be interviewed. Sampled children in mother–child pairs included children under five years of age only. The Gambia 2019–20 DHS reported a 97.0% interview response rate for households, 95.0% response rate for women, and 87.0% response rate among men. The total number of observations in the dataset was 8362 [2]. The analytical sample for this study was 1326 mother–child pairs, after excluding women with no children under 5 years of age (*n* = 435), mother–child pairs missing fever status (*n* = 157), and mothers who reported no child under five years of age with fever in the two weeks before the survey (*n* = 6444).

### Setting

The Gambia is 11295 square kilometres in size. It is situated on the Atlantic Ocean and bordered by Senegal on three sides. The Gambia has limited arable land and is susceptible to drought and flooding, which can severely affect sanitation, health, and nutrition [39]. *P. falciparum* malaria is endemic in The Gambia, with year-round transmission in the western part of the country and seasonal transmission in the Central River and Upper River Regions [40, 41].

The healthcare system is a three-tiered referral system of primary, secondary, and tertiary care. All but one of the five tertiary care government hospitals—Bansang Hospital in the Central River/Janjanbureh Region—are located in the urban Kanifing and Banjul regions [42]. According to the Health Policy Plus project, there were four major and 48 minor health centres, 60 community clinics, and three reproductive and child health centres at the secondary level, and, at the primary level, 634 village health posts in 2019 [43]. Smaller health centres at the secondary level have resident nurses, doctors, and ancillary staff. Government health centres and health posts are located primarily in rural settings [42]. Public sector mobile clinic teams and privately run (non-governmental organization [NGO]) clinics extend the reach. Primary health care at the village level is provided by village health workers and trained midwives. In spite of a commitment to university health coverage, minimal point-of-service fees are assessed for patients over the age of 14 years [43, 44]. Private hospitals/clinics provide care and treatment also, although these are located primarily in Banjul and Kanifing. Informal sources of care and treatment include private pharmacies, shops, markets, itinerant drug sellers, traditional practitioners, or other.

### Variables

Variables in The Gambia 2019–20 DHS child dataset were selected based on a review of public health, socio-behavioural, and anthropological literature on child care-seeking and treatment.

### Outcome variable

The outcome variable is the type of source for care-seeking for a child with fever. Fever is an indicator of malaria and is used as a proxy for suspected malaria in this and other studies using DHS data [45–49]. Mothers who reported a child under 5 years of age ill with a fever in the two weeks prior to the survey were asked where they sought advice or treatment for the sampled child’s fever. Sources of care-seeking for a child with fever have been grouped into four mutually exclusive categories: (1) public facility/provider (i.e., government hospital, health centre, health post, reproductive and child health outreach/mobile clinic, volunteer health worker, and other public health worker); (2) private facility/provider (i.e., private doctor, private hospital/clinic, mobile clinic, and NGO hospital/clinic), located primarily in urban settings; (3) informal source (i.e., private pharmacy, shop, traditional practitioner, or other); and (4) no treatment (i.e., did not seek treatment and nothing was taken for fever). In the 12 instances in which a respondent reported going to a government hospital and a private pharmacy, and the two instances in which a respondent went to a government health centre and a private pharmacy, the response was coded as a public source of care, to ensure mutually exclusive categories of care and maintain the disaggregation of care from a private pharmacy only versus public sources of care. There was no overlap between public and private care sources, or between private pharmacy and other private sector facilities.

The multinomial logistic regression analysis juxtaposes public and private care providers (i.e., trained medical providers) versus no treatment, and public and private care providers versus treatment from private pharmacies, shops, markets, itinerant drug peddlers, and/or traditional healers (a categorization employed by Badolo and colleagues [50]). By disaggregating private pharmacies from private sector (including NGO) hospitals and clinics, doctors, and mobile clinics, factors associated with care-seeking directly from a private pharmacy (regulated) and/or unregulated sources of medicine (such as a shop, market, itinerant drug peddler, or traditional practitioner) can be assessed. The question of interest is not in regard to the skills of private pharmacy staff but rather, the behaviour of care-seekers and what this tells us about challenges they face or their choice and preference in seeking care.

### Explanatory variables

#### Residence

Residence has been categorized as rural or urban by the DHS program and was based on the updated version of the 2013 Gambia Population and Housing Census [51]. In recoding this variable, indeterminate or irreconcilable responses were coded as missing, resulting in 3.4% of observations missing.

#### Household wealth

Household wealth is based on the household wealth index quintiles provided in the DHS dataset, which was derived from a principal component analysis of measures of a household’s cumulative living standard (including consumer goods and housing characteristics in the DHS Phase 7 core questionnaire), adjusted for rural and urban settings [52]. In this study, the two lowest household wealth quintiles (first [poorest] and second quintiles) were grouped together and the upper three wealth quintiles (middle, fourth, and fifth quintiles) grouped together. This division reflects roughly equivalent proportions of Gambian households living in multidimensional poverty (41.7%) [53] versus households living above the poverty line.

#### Mother’s education

Mother’s education was tabulated from a question that asked respondents the highest level of education attended and completed (early childhood education, primary, lower secondary, upper secondary, vocational, diploma, or higher). In this study, mother’s educational level has been recoded as: no education or never completed primary; completed primary but not secondary; or completed secondary or higher.

#### Child’s age

Child’s age in months was recoded into a binary variable (0–23 and 24–59 months) to reflect the standard cut-point for child anthropometry measurement of length (horizontal/supine) or height (standing).

### Covariates

The number of children under five years of age in the household (an ordinal variable) was selected as a covariate based on its use in the literature and its statistical association with the outcome variable. Other potential covariates were assessed but ultimately not selected due to: no association with the outcome variable (i.e., Wald chi-square *p* > 0.05); missingness greater than 10.0%; redundancy (measuring the same thing as a selected variable); or unequal distribution leading to extremely small cell sizes. These included variables measuring a mother’s perceived challenges to seeking medical care for self (distance to health facility, needing money, needing permission, not wanting to go alone) and decision-making (who usually decides on mother’s healthcare and how to spend mother’s earnings [missing 50.6%]), as well as other mother, child, and household characteristics (including mother’s age, child’s body mass index [BMI] z-score for age [missing 50.7%], household size).

### Analysis

Based on the literature, the hypothesis was that household wealth (as a measure of access to financial resources for direct or point of service costs, and indirect costs such as transportation and opportunity costs) would be a significant predictor of fever care-seeking. The question is whether other factors related to the ability to access a health facility or provider might have an equal if not stronger association with care-seeking than household wealth.

Having identified candidate variables following the univariate analysis, bivariate assessments were done to identify variables to include in the initial full model. Model selection employed purposeful selection [54] using backward stepwise regression followed by a reintroduction of each variable individually. The final models, shown below, included the following variables: type of care-seeking source (i.e., the outcome variable that grouped mothers’ responses into public, private, informal, or no treatment); residence (rural or urban); household wealth (two lowest or three highest wealth quintiles); mother’s education (none/some, completed primary, or secondary or higher); child’s age in months (0–23 or 24–59 months); and number of children under 5 years in the household. The first model analysed the likelihood of seeking care from: (1) a public provider versus no treatment; (2) a private provider versus no treatment; and (3) an informal source versus no treatment, according to residence, household wealth, mother’s education, and child’s age, controlling for the number of children under five in the household.

$$log\left( {\frac{{p({\text{care = public)}}}}{{p\left( {{\text{care = none}}} \right)}}} \right) = \beta _{{10}} + ~\beta _{{11}} ({\text{residence = urban)~ + ~}}\beta _{{12}} ({\text{HHW = high)~ + }}~\beta _{{13}} ({\text{mother's~educ = primary)}}$$

$$+ ~\beta _{{14}} ({\text{mother's~educ = secondary~or~higher)~ + ~}}\beta _{{15}} ({\text{child's~age = 24 - 59~mo}}{\text{.)~ + ~}}\beta _{{16}} ({\text{children~ < 5~in~HH)}}$$

$$log\left( {\frac{{p({\text{care = private)}}}}{{p\left( {{\text{care = none}}} \right)}}} \right) = \beta _{{20}} + ~\beta _{{21}} ({\text{residence = urban)~ + ~}}\beta _{{22}} ({\text{HHW = high)~ + }}~\beta _{{23}} ({\text{mother's~educ = primary)}}$$

$$+ ~\beta _{{24}} ({\text{mother's~educ = secondary~or~higher)~ + ~}}\beta _{{25}} ({\text{child's~age = 24 - 59~mo}}{\text{.)~ + ~}}\beta _{{26}} ({\text{children~ < 5~in~HH)}}$$

$$log\left( {\frac{{p({\text{care = informal)}}}}{{p\left( {{\text{care = none}}} \right)}}} \right) = ~\beta _{{30}} + ~\beta _{{31}} ({\text{residence = urban)~ + ~}}\beta _{{32}} ({\text{HHW = high)~}} + ~\beta _{{33}} ({\text{mother's~educ = primary)}}\,$$

$$+ + ~\beta _{{34}} ({\text{mother's~educ = secondary~or~higher)~ + ~}}\beta _{{35}} ({\text{child}}^{{\text{'}}} {\text{s~age = 24 - 59~mo}}{\text{.)~ + ~}}\beta _{{36}} ({\text{children~ < 5~in~HH)}}$$

The second model analysed the likelihood of seeking care from a public provider versus informal source, and a private provider versus informal source, according to residence, household wealth, mother’s education, and child’s age, controlling for the number of children under five in the household.

$$log\left( {\frac{{p({\text{care = public)}}}}{{p\left( {{\text{care = informal}}} \right)}}} \right) = \beta _{{40}} + ~\beta _{{41}} ({\text{residence = urban)~ + ~}}\beta _{{42}} ({\text{HHW = high)~ + }}~\beta _{{43}} ({\text{mother's~educ = primary)}}\,$$

$$+ ~\beta _{{44}} ({\text{mother's~educ = secondary~or~higher)~ + ~}}\beta _{{45}} ({\text{child's~age = 24 - 59~mo}}{\text{.)~ + ~}}\beta _{{46}} ({\text{children~ < 5~in~HH)}}$$

$$log\left( {\frac{{p({\text{care = private)}}}}{{p\left( {{\text{care = informal}}} \right)}}} \right) = \beta _{{50}} + ~\beta _{{51}} ({\text{residence = urban)~ + ~}}\beta _{{52}} ({\text{HHW = high)~ + }}~\beta _{{53}} ({\text{mother's~educ = primary)}}\,$$

$$+ ~\beta _{{54}} ({\text{mother's~educ = secondary~or~higher)~ + ~}}\beta _{{55}} ({\text{child's~age = 24 - 59~mo}}{\text{.)~ + ~}}\beta _{{56}} ({\text{children~ < 5~in~HH)}}$$

A third model analysed the likelihood of seeking care from a public provider versus private provider, according to residence, household wealth, mother’s education, and child’s age, controlling for the number of children under five in the household.

$$log\left( {\frac{{p({\text{care = public)}}}}{{p\left( {{\text{care = private}}} \right)}}} \right) = \beta _{{60}} + ~\beta _{{61}} ({\text{residence = urban)~ + ~}}\beta _{{62}} ({\text{HHW = high)~ + }}~\beta _{{63}} ({\text{mother's~educ = primary)}}\, + ~\beta _{{64}} ({\text{mother's~educ = secondary~or~higher)~ + ~}}\beta _{{65}} ({\text{child's~age = 24 - 59~mo}}{\text{.)~ + ~}}\beta _{{66}} ({\text{children~ < 5~in~HH)}}$$

Descriptive and multinomial logistic regression analyses incorporated sampling strata, primary sampling unit, and women’s individual sample weight provided in the DHS dataset. Multinomial logistic regression analysis generated crude and adjusted odds ratios, statistical significance, and confidence intervals. All proportions reported are weighted proportions. All statistical analyses were performed using SAS statistical software, version 9.4 [55].

## Results

### Characteristics of mothers’ care-seeking for a child with fever; descriptive analysis

Only 52.0% of mothers sought care from a trained healthcare provider for a child under five years of age with a fever—45.1% sought care from a public health facility or provider and 7.0% sought care from a private facility or provider (Table 1). 12.8% of mothers sought treatment from an informal source. Of mothers who sought treatment from an informal source, 92.6% sought treatment from a private pharmacy. Fourteen respondents who sought care from a public provider (12 from a government hospital and 2 from a government health centre) also sought treatment from a private pharmacy. Just over a third of mothers (35.2%) did not seek treatment. The proportion of those who did not seek care from any source plus those who sought care from an informal source was 48.0%.

**Table 1:** Prevalence of child fever care-seeking by source and mother, child, and household characteristics

	Care from a public provider^a^		Care from private provider^b^		Care from an informal source^c^		Did not seek care/treatment		Wald chi-sq
	*n* = 661, 45.07%^d^		*n* = 62, 6.96%^d^		*n* = 140, 12.77%^d^		*n* = 463, 35.21%^d^		
	*n*^e^	%^d^	*n*^e^	%^d^	*n*^e^	%^d^	*n*^e^	%^d^	*p*-value
Region									n/a
Banjul	50	45.19	3	2.78	13	11.56	44	40.47	
Kanifing	47	33.03	15	10.63	37	24.58	45	31.76	
Brikama	71	40.23	18	9.98	20	11.58	68	38.20	
Mansakonko	86	52.29	5	3.37	6	4.20	66	40.14	
Kerewan	62	67.93	0	–	4	4.79	25	27.28	
Kuntaur	137	55.01	2	0.82	26	9.92	83	34.25	
Janjanbureh	76	47.36	17	9.07	6	3.52	62	40.05	
Basse	132	55.40	2	1.11	28	12.21	70	31.28	
Residence^f^									< .0001
Rural	391	56.83	20	2.43	40	5.23	252	35.51	
Urban	260	39.98	37	9.38	93	16.57	187	34.07	
HH wealth quintiles									0.0169
2 lowest	299	46.63	17	5.31	51	8.64	222	39.42	
3 highest	362	43.92	45	8.16	89	15.79	241	32.13	
Needing money for care for self									0.6306
Not a problem	470	44.90	49	7.60	107	13.10	314	34.40	
A big problem	191	45.52	13	5.19	33	11.87	149	37.41	
Perceived access as distance									0.3869
Not a problem	456	46.01	43	7.54	95	13.25	281	33.21	
A big problem	205	42.89	19	5.61	45	11.66	182	39.84	
Mother’s education									0.0032
None/some	361	48.73	23	5.03	56	8.18	256	38.06	
Primary	138	47.79	12	8.25	32	16.64	73	27.32	
Secondary or higher	162	38.46	27	8.98	52	17.17	134	35.40	
Child’s age in months									0.0831
0–23 months	382	45.05	32	6.34	64	10.54	267	38.07	
24–59 months	279	45.09	30	7.75	76	15.65	196	31.52	
Under-5 s in HH									
*n*-size and mean	661	3.60	62	3.73	140	2.57	463	3.29	0.0002

*DHS*, Demographic and Health Survey; *HH*, Household; *NGO*, nongovernmental organization

^a^Public sources included: government hospital; government health centre; government health post; reproductive and child health outreach clinic

^b^Private sector health services included: private hospital/clinic; private doctor; mobile clinic; and NGO hospital/clinic

^c^Informal sources of treatment included: private pharmacy; shop; traditional practitioner; and “other.”

^d^Unweighted sample size

^e^Weighted row proportions

^f^Residence variable is missing 3.4% of observations due to indeterminate or irreconcilable responses

There were significant differences (Wald chi-square *p* < 0.0001) in care-seeking proportions across public, private, or informal source, according to residence (Table 1). A larger proportion of rural residents sought care for a child with fever from a public provider as compared with urban residents (56.8% to 40.0%), and a larger proportion of urban residents sought care from an informal source (primarily a private pharmacy) as compared with rural residents (16.6% to 5.2%). These differences likely reflect the location and accessibility of healthcare options in rural versus urban settings. Proportions of mothers who reported not seeking treatment for a child with fever were similar for rural and urban mothers (35.5% and 34.1%, respectively).

A larger proportion of mothers in households in the upper wealth quintiles sought care from an informal source than mothers in poorer households (15.8% compared with 8.6%) (Table 1). The proportions of mothers with a primary education and mothers with a secondary education who sought care from an informal source were twice that of mothers with little or no education (16.6 and 17.2% compared to 8.2%, respectively) (Table 1); however, these proportion estimates are based on small sample sizes and may be statistically unreliable given large standard errors.

### Multinomial logistic regression results

The logistic regression analysis shows both crude and adjusted odds ratios. Crude odds ratios (cORs) are reported in Table 2. Mothers in urban households were four times as likely as mothers in rural households to seek care for a child with fever from a private provider versus not seeking treatment (adjusted odds ratio [aOR] 4.20, 95% CI 1.472–12.001; although this estimate may be unreliable due to small sample sizes and large standard error), and almost three times as likely as mothers in rural households to seek care from an informal source versus not seeking treatment (aOR 2.67, 95% CI 1.504–4.736, Wald Chi-square *p* < 0.001), after adjusting for covariates (Table 3). Mothers in households in the upper wealth quintiles were twice as likely as mothers in the lowest wealth quintiles to seek care from an informal source versus not seeking treatment (aOR 2.30, 95% CI 1.274–4.164, Wald Chi-square *p* < 0.01), after adjusting for covariates. Mothers who completed a primary education were more likely than mothers with little or no education to seek care for a child with fever from a private health provider versus no treatment (aOR 2.39, 95% CI 1.086–5.237), and more likely to seek care from an informal source versus no treatment than mothers with little or no education (aOR 2.64, 95% CI 1.352–5.170) (Table 3) after adjusting for covariates, although these estimates may be unreliable due to small sample sizes and large standard errors.

**Table 2:** Logistic regression analysis results: Crude odds ratios, child fever care-seeking

	*n*^a^	cOR	95% CI	*n*^a^	cOR	95% CI	*n*^a^	cOR	95% CI
	Care from a public provider^b^ vs. no treatment			Care from private provider^c^ vs. no treatment			Care from an informal source^d^ vs. no treatment		
	*n* = 661, 45.07%^e^			*n* = 62, 6.96%^e^			*n* = 140, 12.77%^e^		
Residence^f^									
Rural	391	1.0 (ref)	–	20	1.0 (ref)	–	40	1.0 (ref)	–
Urban	260	0.73	0.523–1.029	37	4.03*^g^	1.323–12.263	93	3.30****	1.887–5.780
HH wealth quintiles									
2 lowest	299	1.0 (ref)	–	17	1.0 (ref)	–	51	1.0 (ref)	–
3 highest	362	1.16	0.809–1.652	45	1.89	0.930–3.827	89	2.24**	1.332–3.772
Mother’s education									
None/some	361	1.0 (ref)	–	23	1.0 (ref)	–	56	1.0 (ref)	–
Primary	138	1.37	0.904–2.063	12	2.29*^g^	1.074–4.864	32	2.83**^g^	1.475–5.435
Secondary or higher	162	0.85	0.599–1.202	27	1.92*	1.005–3.669	52	2.26**	1.363–3.731
Child’s age in months									
0 to 23 months	382	1.0 (ref)	–	32	1.0 (ref)	–	64	1.0 (ref)	–
24 to 59 months	279	1.21	0.839–1.741	30	1.48	0.797–2.726	76	1.79*	1.129–2.850
Under-5 s in HH^h^	661	1.05	0.987–1.116	62	1.07	0.934–1.220	140	0.84**	0.739–0.959

	Care from a public provider^b^ vs. informal source^d^			Care from private provider^c^ vs. informal source^d^					
Residence^f^									
Rural	391	1.0 (ref)	–	20	1.0 (ref)	–			
Urban	260	0.22****	0.129–0.382	37	1.22	0.373–3.982			
HH wealth quintile									
2 lowest quintiles	299	1.0 (ref)	–	17	1.0 (ref)	–			
3 highest quintiles	362	0.52*	0.307–0.867	45	0.84*	0.385–1.840			
Mother’s education									
None/some	361	1.0 (ref)	–	23	1.0 (ref)	–			
Primary	138	0.48*^g^	0.255–0.913	12	0.81	0.320–2.039			
Secondary/higher	162	0.38***	0.214–0.662	27	0.85	0.382–1.898			
Child’s age									
0 to 23 months	382	1.0 (ref)	–	32	1.0 (ref)	–			
24 to 59 months	279	0.67	0.434–1.048	30	0.82	0.413–1.637			
Children < 5 yrs in HH^h^	661	1.25***	1.108–1.404	62	1.27**	1.072–1.501			

	Care from a public provider^b^ vs. private provider^c^								
Residence^f^									
Rural	391	1.0 (ref)	–						
Urban	260	0.18** ^g^	0.059–0.562						
HH wealth quintile									
2 lowest quintiles	299	1.0 (ref)	–						
3 highest quintiles	362	0.61	0.298–1.258						
Mother's education									
None/some	361	1.0 (ref)	–						
Primary	138	0.60	0.264–1.352						
Secondary/higher	162	0.44* ^g^	0.216–0.905						
Child's age									
0 to 23 months	382	1.0 (ref)	–						
24 to 59 months	279	0.82	0.433–1.552						
Children < 5 yrs in HH^h^	661	0.98	0.858–1.127						

*p < 0.05; **p < 0.01; ***p < 0.001; ****p < 0.0001

*DHS*, Demographic and Health Survey; *HH*, Household; Under-5 s, children under 5 years of age; *NGO*, nongovernmental organization

^a^Unweighted sample size

^b^Public sources included: government hospital; government health centre; government health post; and reproductive and child health outreach [mobile] clinic

^c^Private sector health services included: private hospital/clinic; private doctor; mobile clinic; and NGO hospital/clinic

^d^Informal sources of treatment included: private pharmacy; shop; traditional practitioner; and “other.”

^e^Weighted proportion

^f^Residence variable is missing 3.4% of observations due to indeterminate or irreconcilable responses

^g^May be statistically unreliable due to small sample sizes and percent/standard error > 30%

^h^The number of children under 5 years of age in the household is an ordinal variable

**Table 3:** Logistic regression analysis results: Adjusted odds ratios, child fever care-seeking

	*n*^a^	aOR	95% CI	*n*^a^	aOR	95% CI	*n*^a^	aOR	95% CI
	Care from a public provider^b^ vs. no treatment			Care from private provider^c^ vs. no treatment			Care from an informal source^d^ vs. no treatment		
	*n* = 661, 45.07%^e^			*n* = 62, 6.96%^e^			*n* = 140, 12.77%^e^		
Residence^f^									
Rural	391	1.0 (ref)	–	20	1.0 (ref)	–	40	1.0 (ref)	–
Urban	260	0.78	0.546–1.110	37	4.20** ^g^	1.472–12.001	93	2.67***	1.504–4.736
HH wealth quintiles									
2 lowest	299	1.0 (ref)	–	17	1.0 (ref)	–	51	1.0 (ref)	
3 highest	362	1.22	0.846–1.752	45	1.79	0.831–3.859	89	2.30**	1.274–4.164
Mother’s education									
None/some	361	1.0 (ref)	–	23	1.0 (ref)	–	56	1.0 (ref)	
Primary	138	1.50	0.979–2.284	12	2.39* ^g^	1.086–5.237	32	2.64** ^g^	1.352–5.170
Secondary or higher	162	0.87	0.589–1.292	27	1.38	0.715–2.680	52	1.59	0.883–2.852
Child’s age in months									
0 to 23 months	382	1.0 (ref)	–	32	1.0 (ref)	–	64	1.0 (ref)	
24 to 59 months	279	1.21	0.829–1.768	30	1.51	0.785–2.906	76	1.83*	1.094–3.046
Under-5 s in HH^h^	661	1.03	0.964–1.101	62	1.10	0.966–1.244	140	0.88*	0.777–0.998

	Care from a public provider^b^ vs. informal source^d^			Care from private provider^c^ vs. informal source^d^					
Residence^f^									
Rural	391	1.0 (ref)	–	20	1.0 (ref)	–			
Urban	260	0.29****	0.165–0.515	37	1.58	0.501–4.949			
HH wealth quintiles									
2 lowest	299	1.0 (ref)	–	17	1.0 (ref)	–			
3 highest	362	0.53*	0.291–0.959	45	0.78	0.352–1.716			
Mother’s education									
None/some	361	1.0 (ref)	–	23	1.0 (ref)	–			
Primary	138	0.57	0.287–1.114	12	0.90	0.346–2.355			
Secondary or higher	162	0.55	0.280–1.079	27	0.87	0.375–2.028			
Child’s age in months									
0 to 23 months	382	1.0 (ref)	–	32	1.0 (ref)	–			
24 to 59 months	279	0.66	0.410–1.071	30	0.83	0.406–1.687			
Under-5 s in HH^h^	661	1.17*	1.037–1.320	62	1.25**	1.058–1.466			

	Care from a public provider^b^ vs. private provider^c^								
Residence^f^									
Rural	391	1.0 (ref)	–						
Urban	260	0.19** ^g^	0.063–0.544						
HH wealth quintiles									
2 lowest	299	1.0 (ref)	–						
3 highest	362	0.68	0.313–1.478						
Mother’s education									
None/some	361	1.0 (ref)	–						
Primary	138	0.63	0.264–1.488						
Secondary or higher	162	0.63	0.296–1.342						
Child’s age in months									
0 to 23 months	382	1.0 (ref)	–						
24 to 59 months	279	0.80	0.410–1.565						
Under-5 s in HH^h^	661	0.94	0.816–1.082						

*p < 0.05; **p < 0.01; ***p < 0.001; ****p < 0.0001

DHS, Demographic and Health Survey; HH, Household; Under-5 s, children under 5 years of age; NGO, nongovernmental organization

^a^Unweighted sample size

^b^Public sources included: government hospital; government health centre; government health post; and reproductive and child health outreach [mobile] clinic

^c^Private sector health services included: private hospital/clinic; private doctor; mobile clinic; and NGO hospital/clinic

^d^Informal sources of treatment included: private pharmacy; shop; traditional practitioner; and “other.”

^e^Weighted proportion

^f^Residence variable is missing 3.4% of observations due to indeterminate or irreconcilable responses

^g^May be statistically unreliable due to small sample sizes and percent/standard error > 30%

^h^The number of children under 5 years of age in the household is an ordinal variable

Mothers in urban households were 0.29 times as likely (aOR, 95% CI 0.165–0.515) than mothers in rural households to seek care from a public provider versus informal source, after adjusting for covariates. Mothers in households in the higher wealth quintiles were half as likely as mothers in poorer households to seek care from a public provider versus informal source (aOR 0.53, 95% CI 0.291–0.959), after adjusting for covariates. Mothers in urban households were 0.19 times as likely than mothers in rural households to seek care from a public versus private provider (aOR 0.19, 95% CI 0.063–0.544) (Table 3), after adjusting for covariates, although this estimate may be unreliable due to small sample sizes and large standard error.

## Discussion

Previous studies have found associations between child care- and treatment-seeking choices and: household wealth; direct costs (i.e., point of service fees); indirect costs (e.g., transportation); and/or opportunity costs (e.g., the cost of time away from farming and household chores) of healthcare [45–50, 56, 57]. Associations between healthcare costs, having money, and seeking care are not always easy to interpret, however. Contrary to the initial hypothesis, this analysis of The Gambia 2019–20 DHS did not find evidence that greater household wealth is associated with increased odds of seeking care for a child with fever from either a public or private health facility versus no treatment. Instead, greater household wealth was associated with seeking treatment from an informal source versus no treatment. User fees at public health facilities are exempt for mothers and children under five years of age in The Gambia [43], which should mitigate financial barriers to the use of public facilities. This does not explain the association between household wealth and care-seeking from an informal source.

A recent study on costs and barriers to seeking malaria treatment in the Upper River Region of The Gambia by Broekhuizen and colleagues [58] found that regular stock-outs of drugs for malaria treatment at health facilities contributed to the high costs of healthcare services reported. As a result of stock-outs, care-seekers had to purchase malaria treatment from private pharmacies. An analysis of DHS data collected in the 1990s from 14 countries, by Filmer et al*.* [45] also found that the use of a pharmacy or shop for treatment for a child with fever in rural areas was significantly associated with greater household wealth. A multi-country analysis of DHS and Malaria Indicator Survey data by Shah et al*.* [48] found that care-givers in households in the lowest wealth quintile sought out free artemisinin-based combination therapy (ACT; a recommended treatment for uncomplicated *P. falciparum* malaria) from public sector facilities/providers, while care-givers from households with greater wealth sought first-line ACT from private retailers. These studies appear to suggest that, with greater household wealth comes an ability to seek malaria care and treatment from additional options within a pluralistic healthcare setting. More healthcare options are available in urban settings, although care from a private hospital/clinic comes at a greater cost. Treatment from a shop or unregulated pharmacy come at greater risk due to questionable quality of appropriateness of drugs for purchase, but may be perceived as having greater flexibility and convenience than seeking care from a public healthcare source.

A systematic review conducted by Colvin et al*.* [34] to understand care-seeking for child illness in sub-Saharan Africa uncovered convenience and economic incentives as reasons for seeking treatment from pharmacies, local drug sellers, or other local retailers. More convenient access (for urban residents in particular) because of distance to home, longer hours of operation, and faster service, the option of receiving treatment on credit, and an ability to purchase treatment in varying quantities, depending on what a care-seeker could afford at the time, has been reported as an important consideration [59–64], even though this is not optimal from a bio-medical standpoint. Private pharmacies are mostly located in urban areas and rarely found in rural areas in The Gambia, according to the African Health Workforce Observatory [65]. Mothers in households with greater wealth, and in households in urban areas are more likely to have the option of going to a private pharmacy, where the hours of operation, shorter distances, or wait-times could be perceived as more accessible, convenient and accommodating to their needs [66, 67].

While this study did not find an association between care-seeking source and access (perceived distance to a health facility), other studies have noted distance and accessibility as a factor in care-seeking decisions. For example, a study examining care-seeking choices for a child with fever in Malawi [47] found that care-givers in urban households were more likely to go to a hospital versus a traditional provider or not seeking care for a child with fever; however, care-givers who reported time to a facility as a big problem, or transport availability as a big problem, were less likely to seek care from a hospital versus other care. Convenience and affordability have emerged as incentives to go to medicine sellers in other studies as well [68–70], although when and where to seek care varied according to the immediate context and perceived need [71]. A study on rural–urban disparities in malaria care-seeking carried out in Kaduna (northwestern Nigeria) by Babalola et al*.* [72] found that mothers residing in rural settlements were significantly more likely to delay care-seeking but less likely to seek care from sources such as a private pharmacy or patent medicine vendor for a child with fever.

Various studies have identified less easily measurable, but nonetheless important factors that influence child fever care-seeking behaviour. Perceptions of malaria cause(s), severity, and perceived treatment effectiveness or relevance can be strong influences on care-seeking [24, 59, 62, 72, 73]. A fever caused by a spirit or malicious intent could indicate an illness not treatable by a biomedically trained health provider [74]. Urgency or a lack of urgency (e.g., waiting to see if a fever goes away) as well as convenience, particularly when medicines can be purchased quickly and easily from a drug seller or pharmacy also influence care-seeking decisions [22, 24, 72, 75]. Perceived urgency, convenience, and previous experiences in seeking treatment for a child with fever may explain the increased likelihood that mothers of children 24 to 59 months in The Gambia sought treatment for the child’s fever from an informal source.

Care-seeking decisions are complex. Strategies for mitigating cost [20] and the perceived advantages or risks of going directly to a pharmacy or other drug vendor are part of the decision-making process that includes a host of competing [76] and often hidden priorities as well as other factors embedded in a mother’s and household’s social ecology [77]. Various scholars have addressed the role of women’s autonomy in care-seeking [78–82], although measures of decision-making autonomy were not associated with care outcomes in this study. Individually-perceived ability and the availability, access, cost, and convenience of care- and treatment-seeking options are difficult to untangle from the hidden influences on decision-making.

These findings have implications for the care of children with fever in The Gambia. Whether related to convenience, perceived urgency, previous experience, or other influences, mothers in urban residences and mothers in wealthier households were more likely to seek treatment from an informal source than mothers in rural residences or poorer households. This illustrates a clear demand, especially for families in urban areas and market centres, for alternative sources, such as retail pharmacies and medicine shops, for treatment for a child with fever and suspected malaria. Meeting this demand will require more engagement of private pharmacies in malaria control strategies—a suggestion that has been made by various scholars [64, 70, 75, 84]—and additional funding and a commitment to adequate oversight by The Gambia Pharmacy Council and Medicine Control Agency to ensure compliance with pharmaceutical sector regulations [43]. Maintenance of the public sector health workforce is also needed, given an environment where trained medical staff find better paying options in the non-governmental sector [85], and provide accessible, appropriate care through integrated community case management of malaria and other childhood illnesses [64, 86].

In addition, it is necessary to explore the contextually-situated complexity of individual and household decision-making to address behavioural change. Quantitative data available in the DHS are not sufficient to explain why, in The Gambia, nearly half of mothers (48.0%) did not seek care for a child with fever from a trained healthcare provider. Household wealth or the ability to afford optimal care is not sufficient to explain care-seeking without considering how it acts together with other factors. Further directions for research, with the aim of informing the interpretation and application of findings from this study might include: a comparison with similar studies of other country DHS data; and qualitative, field-based research to uncover emic perceptions and underlying behavioural constructs [87] that influence child fever care-seeking.

### Strengths and limitations

This study is unique in the analysis of recent, nationally representative data to look specifically at child fever care-seeking in The Gambia, by grouping responses into public, private, and informal sources of care. A particular strength of this study is the disaggregation of private pharmacy treatment-seeking from private care sources, and an analysis of trained care providers disaggregated by public and private providers. As a result of this disaggregation, this analysis was able to explore factors influencing child fever care-seeking behaviour with greater nuance than would otherwise be possible.

This study relied on secondary data for analysis and did not include additional tests of validity and reliability of mothers’ responses. Given limitations to self-reported data, there are challenges to internal validity due to potential response bias, desirability bias, and concerns with cross-cultural construct validity. Using fever as a proxy for malaria is a limitation of this study as well. In sub-Saharan Africa, the proportion of fever cases documented with malaria parasitaemia ranges from 2.0% to 81.0% [88]. DHS reliance on care-giver responses and the 2 week recall period likely underestimates disease incidence [89] while potentially overestimating malaria incidence. Urban/rural residence classifications may limit the interpretation to some extent since they are based on government administrative aspects and do not necessarily capture the diversity of localized, social, and built environments [90]. The relatively small sample size (*n* = 1326) may have limited the ability to detect associations and contributed to unreliable estimates of care-seeking from private care sources when disaggregated from public sources.

## Conclusions

Without appropriate care, The Gambia’s achievements in malaria control could stall, thus slowing progress toward the sustainable development communicable diseases goal (SGD 3.3; ‘ending the epidemics of AIDS, tuberculosis, malaria and neglected tropical diseases and combat hepatitis, water-borne diseases and other communicable diseases’). It will be crucial to reach more children with appropriate diagnosis, treatment, and referral, through greater access to appropriate alternative sources of care, a continued expansion of integrated community case management, and more effective attitude and behavioural change strategies for mothers and households for improved care-seeking.

## Data Availability

DHS data are accessible upon request from the DHS Program; https://dhsprogram.com/data/Using-DataSets-for-Analysis.cfm.

## Abbreviations

ACT: Artemisinin-based combination therapy
aOR: Adjusted odds ratio
CI: Confidence interval
cOR: Crude odds ratio
DHS: Demographic and health survey
GBoS: Gambia Bureau of Statistics
HH: Household
HHW: Household wealth
NGO: Non-governmental organization
Under-5 s: Children under 5 years of age
UNDP: United Nations Development Programme
WHO: World Health Organization
